# An Intelligent Energy-Aware Framework for 6G-Enabled Non-Terrestrial IoT via Reinforcement Learning

**DOI:** 10.3390/s26134057

**Published:** 2026-06-26

**Authors:** Ali Nauman, Sung Won Kim

**Affiliations:** School of Computer Science and Engineering, College of Digital Convergence, Yeungnam University, Gyeongsan 38541, Republic of Korea; swon@yu.ac.kr

**Keywords:** 6G, Internet of Things (IoT), Q-learning, Reinforcement Learning (RL), energy efficiency (EE), Non-Terrestrial Networks (NTNs)

## Abstract

6G promises ultra-low latency, high data throughput, and seamless global connectivity. However, providing uninterrupted connectivity in remote and underserved regions remains a critical challenge for Terrestrial Networks (TNs), where the cost of deploying infrastructure is difficult to justify against sparse user density. Standardized under 3GPP Release 17, Non-Terrestrial Networks (NTNs) have emerged as a viable solution to close this digital divide. Among NTN platforms, High-Altitude Platform Stations (HAPS) occupy a strategic middle ground, as they deliver lower propagation delays than Low-Earth Orbit (LEO) satellites while achieving far broader coverage than TN-based Base Stations (BS). Despite these advantages, battery-powered Internet of Things (IoT) devices communicating via HAPS face a fundamental energy efficiency (EE) challenge: transmit power must be carefully managed to maximize data throughput while preserving battery life and minimizing packet queuing delays. To address this, we propose a Q-learning-based Reinforcement Learning (RL) framework. The RL agent observes the instantaneous battery level and queue state of the IoT device, and dynamically selects optimal power levels from a discrete action space across successive time slots. Unlike traditional heuristic algorithms, such as Round Robin (RR), Max Single-to-Noise Ratio (Max-SNR), and fixed-power allocation, which rely on static rules or greedy channel-based decisions, the proposed Q-learning agent learns adaptive, long-term optimal policies through direct interaction with the environment, without requiring explicit mathematical modeling of the channel or traffic dynamics. Extensive simulations demonstrate that the proposed framework achieves up to 40% higher average EE compared to all benchmark schemes, maintains consistently lower power consumption, and exhibits superior statistical reliability as evidenced by a right-shifted Cumulative Distribution Function (CDF) of EE. These results demonstrate Q-learning as a promising candidate for scalable, energy-aware power control of next-generation HAPS-assisted IoT deployments in 6G NTN ecosystems.

## 1. Introduction

The rapid evolution of wireless communications toward 6G networks demands ubiquitous connectivity, ultra-reliable low-latency communications, and massive Internet of Things (IoT) support [1,2,3]. To meet these goals, future architectures integrate terrestrial and non-terrestrial segments into Vertical Heterogeneous Networks (VHetNets) [4], with Non-Terrestrial Networks (NTN) playing a key role in extending coverage to remote and disaster-affected regions where conventional infrastructure is unavailable or uneconomical [5].

Among NTN technologies, High-Altitude Platform Stations (HAPS) have gained considerable attention due to their advantageous operational characteristics [6]. Typically deployed in the stratosphere at altitudes of approximately 17–22 km, HAPS provide wide-area coverage with significantly lower propagation delay than satellite systems, while requiring less dense infrastructure compared to Terrestrial Networks (TN) [7]. These platforms can maintain quasi-stationary positions, enabling stable and continuous service delivery. Recent advances in solar energy harvesting, lightweight composite materials, antenna design, and autonomous flight control have further improved the feasibility and cost-effectiveness of HAPS systems [7]. Moreover, their ability to host edge computing resources allows them to function as aerial data centers, facilitating real-time data processing and reducing backhaul dependency in data-intensive applications such as the IoT [8].

Despite these advantages, several challenges remain in the efficient operation of HAPS-assisted networks, particularly in energy-constrained IoT environments [9]. Battery-powered IoT devices must carefully balance transmit power, data throughput, and latency, especially when communicating over time-varying wireless channels. Traditional resource allocation methods, such as fixed-power schemes, Round Robin (RR) scheduling, and channel-aware greedy approaches (e.g., Max Single-to-Noise Ratio (Max-SNR)), often fail to capture the dynamic nature of wireless environments and typically optimize only a single performance metric [10]. Consequently, they are not well suited for scenarios requiring joint optimization of Energy Efficiency (EE), reliability, and delay.

### 1.1. Motivation

Three converging trends motivate this work. First, connected IoT endpoints are projected to reach 35 billion by 2030 [11], many deployed in areas beyond terrestrial reach. Second, 6G integration of TN and NTN [12] makes global IoT coverage technically feasible for the first time. Third, energy consumption remains the primary bottleneck for battery-powered IoT devices in remote environments where recharging is infeasible [13,14,15].

HAPS platforms sit at a unique intersection of these three trends: they are capable of serving large numbers of IoT devices across wide geographic areas, they are being actively standardized as part of the 6G NTN framework, and they introduce specific challenges related to propagation distance and power management that demand intelligent, adaptive solutions. This motivates the need for an Artificial Intelligence (AI)-driven power control framework that can maximize EE while respecting the practical constraints of battery-operated IoT devices [16].

### 1.2. Challenges

Several technical challenges make the energy-efficient power control problem in HAPS-assisted IoT systems non-trivial:

1. Dynamic Channel Conditions: The wireless channel between IoT devices and HAPS undergoes rapid temporal variations due to atmospheric turbulence, shadowing, and multipath propagation. Static power allocation schemes cannot adapt to these variations, leading to either wasted energy or degraded link quality.
2. Battery Constraints: IoT devices in remote areas are typically constrained by finite battery capacity. Aggressive power allocation may achieve higher instantaneous throughput but at the cost of premature battery depletion, ultimately reducing the operational lifetime of the device.
3. Queue Management: Stochastic packet arrivals create variable traffic loads that must be managed efficiently. Excessive queue buildup leads to increased end-to-end latency, while aggressive queue draining may encourage power waste on transmissions of marginal benefit.
4. Multi-Objective Optimization: Jointly optimizing EE, throughput, and packet delivery ratio under dynamic channel conditions represents a complex, high-dimensional optimization problem that does not admit closed-form solutions in general settings.
5. Model Uncertainty: Accurate mathematical models of the channel, battery dynamics, and traffic arrivals are often unavailable or computationally expensive to maintain in real time. Model-free approaches that can learn directly from environmental interaction are therefore highly desirable.

### 1.3. Contributions

To address these limitations, recent studies have explored the application of AI techniques in HAPS-enabled communication systems. Reinforcement Learning (RL), in particular, has been widely adopted for decision-making problems in wireless networks, including spectrum allocation, handoff management, and user scheduling [17]. Neural network-based methods have also been investigated for mobility prediction and adaptive control, while optimization techniques have been applied to improve beamforming and resource allocation [18,19,20]. Additionally, emerging concepts such as Federated Learning (FL) and distributed AI have been considered for enabling collaborative intelligence across aerial platforms [21]. However, many of these approaches either rely on accurate prior knowledge of the environment or face scalability issues when deployed on resource-limited IoT devices [22]. Furthermore, the integration of energy-aware learning mechanisms tailored for HAPS-assisted IoT communication remains insufficiently explored.

Motivated by these challenges, this paper proposes an intelligent and energy-aware transmit power control framework for IoT devices communicating via HAPS in a 6G NTN environment. The proposed approach leverages RL to enable adaptive and autonomous decision-making under dynamic network conditions. Specifically, the transmit power control problem is formulated as a Markov Decision Process (MDP), where the system state captures both the battery level and traffic queue dynamics of the IoT devices. A Q-learning algorithm is employed to learn optimal power allocation policies through interaction with the environment, eliminating the need for explicit channel modeling.

The main contributions of this work are summarized as follows:

1. A comprehensive framework is developed that jointly considers channel variability, battery constraints, and traffic dynamics in HAPS-assisted IoT communication. The model captures the interdependencies between these components and serves as the foundation for RL-based optimization.
2. The transmit power control problem is formulated as an MDP and solved using a Q-learning approach that enables adaptive and model-free decision-making. The agent interacts directly with the system environment, learning optimal policies without requiring explicit knowledge of channel statistics or traffic patterns.
3. A carefully designed reward function balances EE, queueing delay, and packet loss, ensuring practical applicability in real-world IoT scenarios. The reward formulation encodes the trade-offs among competing objectives and guides the RL agent toward policies that are beneficial in the long run.
4. Extensive simulations demonstrate that the proposed method significantly outperforms conventional schemes, including random power selection, RR, Max-SNR, and fixed-power allocation, achieving higher EE, lower power consumption, and more stable performance. The superiority of the proposed approach is further confirmed by statistical analysis using Cumulative Distribution Function (CDF).
5. The computational complexity of the Q-learning agent is analyzed with respect to the state and action space sizes, demonstrating that the proposed framework scales gracefully and is deployable on resource-constrained IoT hardware.

While Q-learning has been broadly applied to wireless resource management [17], to the best of authors’ knowledge, this work combines three design choices not previously unified in the HAPS-IoT literature: (i) a *joint battery-queue state space* $s\left(t\right)=[\hat{B}\left(t\right),\;\hat{Q}\left(t\right)]$ that simultaneously captures energy longevity and latency; prior HAPS-RL studies typically optimize a single dimension; (ii) a *lightweight tabular formulation* with |S|×|A|=400 entries, deliberately chosen to remain within the compute and memory budget of Class-AB IoT transceivers, in contrast to Deep Q-learning (DQN)-based approaches that require neural network inference engines; and (iii) a *multi-objective composite reward* $r\left(t\right)=\beta_{1}\cdot \mathrm{EE}\left(t\right)-\beta_{2}\cdot Q\left(t\right)-\beta_{3}\cdot \mathrm{Drop}\left(t\right)$, tailored for the HAPS uplink where long propagation paths make retransmissions prohibitively costly.

### 1.4. Related Work

Prior RL-based power control studies fall into three categories. (a) *Terrestrial RL power control*: Works such as Luong et al. [17] optimize spectral efficiency or throughput in cellular networks without battery or propagation-distance constraints. (b) *HAPS resource management without RL*: Optimization-based schemes (e.g., beamforming design [18,20]) achieve strong performance but require accurate channel models and do not adapt to dynamic battery or queue states. (c) *RL in NTN/satellite contexts*: Emerging studies apply DQN to satellite scheduling [23] but focus on multi-user interference rather than single-device energy-latency trade-offs. The present work fills the gap at the intersection of these categories: a hardware-feasible, model-free RL framework for the HAPS-IoT uplink that jointly manages battery depletion, queue dynamics, and EE.

## 2. System Model

### 2.1. Network Architecture

**Figure 1:**
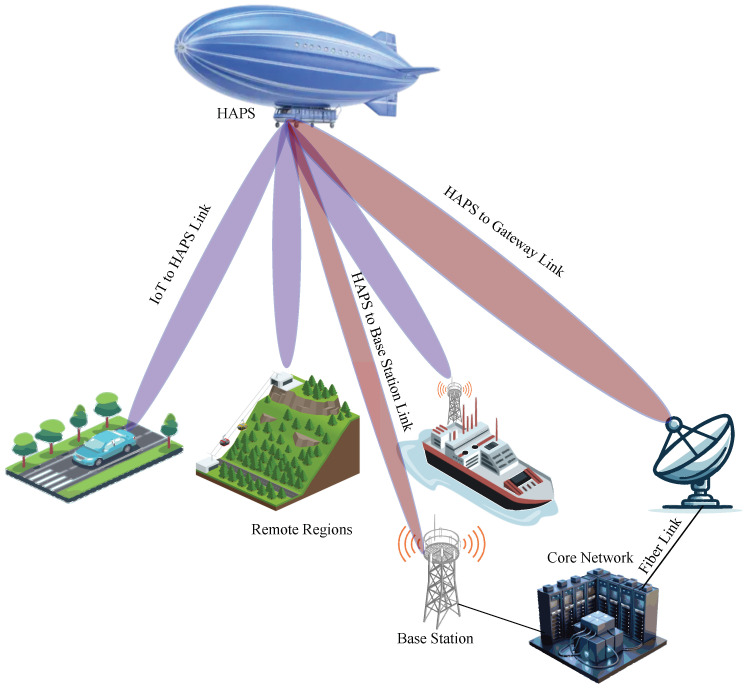
System architecture: IoT device communicating with HAPS relay.

We consider a battery-powered IoT device located in a remote area that communicates wirelessly with a HAPS, as illustrated in Figure 1. The HAPS is deployed in the stratosphere at an altitude of *H* and serves as a relay node, forwarding received IoT data to a terrestrial Base Station (BS) via a high-capacity backhaul link. The BS is connected to the core network and provides gateway services to external networks.

The IoT device is assumed to be stationary or quasi-stationary (e.g., a sensor node mounted on agricultural equipment or a remote monitoring station). The device is powered by a finite-capacity battery and generates packetized data that must be transmitted to the HAPS for further processing and forwarding. The system operates in discrete time slots t=1,2,…,T, where each time slot corresponds to a fixed transmission interval of duration τ seconds.

### 2.2. Channel Model

The wireless channel between the IoT device and the HAPS experiences time-varying fading. Given the quasi-line-of-sight nature of the stratospheric channel and the quasi-stationary position of both the IoT device and the HAPS, the channel gain h(t) at time slot *t* is modeled as a bounded random variable:

(1) h(t)∼U(0,1),

where U(0,1) denotes a uniform distribution on the interval [0,1]. This model captures the aggregate effect of free-space path loss, atmospheric absorption, and small-scale fading, normalized to a unit-maximum gain. Its adoption is intentional for two reasons: (a) it isolates the RL agent’s decision-making behavior from channel-model assumptions, enabling a clean evaluation of the framework; and (b) it represents a conservative, worst-case scenario of maximum channel uncertainty, as the agent cannot exploit any statistical structure in the fading process; a demanding test of robustness.

For practical HAPS stratospheric links, a Rician model with a strong Line-of-Sight (LoS) component (*K*-factor ≥ 10 dB) is more physically accurate. Under such a model, the channel-gain distribution would be more tightly concentrated around its mean, making the agent’s task easier than what we simulate here; U(0,1) therefore provides a performance lower bound for the RL framework. For low-elevation HAPS geometries, the shadowed-Rician (Loo) model captures combined fading and shadowing more faithfully and is recommended in 3GPP TR 38.811 for NTN channel modeling. Substituting any of these models is straightforward given the channel-agnostic structure of the RL framework, and is identified as a concrete future extension. The free-space path loss at the HAPS altitude is given by:

(2) $$L_{\mathrm{fs}}={\left(\frac{4\pi df_{c}}{c}\right)}^{2},$$

where *d* is the slant range between the IoT device and the HAPS, $f_{c}$ is the carrier frequency, and *c* is the speed of light.

### 2.3. Assumptions

The following assumptions are made throughout the paper:

1. The HAPS-to-BS backhaul link has sufficient capacity and is not a bottleneck; hence, we focus exclusively on the IoT-to-HAPS uplink.
2. The IoT device has no knowledge of future channel states; decisions at time *t* are based solely on the current state s(t)=[B(t),Q(t)].
3. Packet arrivals are Independent and Identically Distributed (i.i.d.) across time slots.
4. The RL agent operates on the IoT device and has access only to locally observable quantities (battery level and queue length), making the framework fully distributed.
5. The Q-table is pre-trained in simulation and loaded onto the IoT device for inference; the device does not perform online Q-table updates during deployment.

### 2.4. Communication Model

The received signal-to-noise ratio (SNR) at the HAPS is given by

(3) $$\mathrm{SNR}\left(t\right)=\frac{P(t)\cdot h(t)}{N_{0}},$$

where P(t) is the transmit power at time slot *t*, h(t) is the instantaneous channel gain, and $N_{0}$ is the Additive White Gaussian Noise (AWGN) power spectral density. The achievable transmission rate (in bits per time slot) is given by the Shannon capacity formula:


(4) $$R\left(t\right)=\log_{2}\;\left(1+\frac{P(t)\cdot h(t)}{N_{0}}\right).$$

The transmit power P(t) is selected from a finite discrete set:

(5) P={0,0.1,0.5,1.0}(Watts).

This discrete action space is motivated by practical hardware constraints: many IoT transceivers support only a small number of programmable output power levels. The action P(t)=0 corresponds to a silent time slot (no transmission), which may be chosen by the RL agent when the channel quality is poor or the battery level is critically low.

A transmission at time slot *t* is considered successful if the instantaneous SNR exceeds a predefined threshold $\gamma_{\mathrm{th}}$:

(6) $$S\left(t\right)=\left\{\begin{array}{cc} 1 & \mathrm{if}\;\mathrm{SNR}\left(t\right)\geq \gamma_{\mathrm{th}},\\ 0 & \mathrm{otherwise}. \end{array}\right.$$

When P(t)=0, we set S(t)=0 by definition. The SNR threshold $\gamma_{\mathrm{th}}$ is set to correspond to a minimum required data rate, reflecting the target Quality of Service (QoS) for the IoT application.

To provide context for the numerical parameters used in the simulations, we present a simplified link budget analysis. The received power at the HAPS is

(7) $$P_{\mathrm{rx}}=P\left(t\right)\cdot G_{\mathrm{tx}}\cdot G_{\mathrm{rx}}\cdot h\left(t\right)\cdot L_{\mathrm{fs}}^{-1},$$

where $G_{\mathrm{tx}}$ and $G_{\mathrm{rx}}$ are the transmit and receive antenna gains, respectively. For a typical IoT device with an omnidirectional antenna ($G_{\mathrm{tx}}=0$ dBi) and a HAPS with a directive antenna ($G_{\mathrm{rx}}=30$ dBi), the aggregate gain partially compensates for the large free-space path loss. In the simulation model, these gains are absorbed into the effective channel gain h(t), which is normalized to [0,1].

### 2.5. Energy and Battery Model

The energy consumed for a single transmission at time slot *t* is modeled as

(8) E(t)=η·P(t),

where η is a scaling factor that accounts for the duration of the time slot, the efficiency of the power amplifier, and any additional circuit power overhead. In the simulations, we set η=10, which maps the maximum transmit power of 1 W to an energy expenditure of 10 units per slot. This linear model is a standard approximation for Class-AB power amplifiers used in low-power IoT transceivers.

The IoT device is powered by a finite-capacity battery with maximum energy $B_{\max}$. The battery level evolves according to

(9) $$B\left(t+1\right)=\max\;\left(0,\;B(t)-E(t)\right),$$

where the max(0,·) operator enforces the constraint that the battery cannot be depleted below zero. No energy harvesting is considered in this work; the battery is charged externally and replaced when depleted.

The battery level B(t) is discretized into $N_{B}$ levels for the purpose of RL state representation:

(10) $$\hat{B}\left(t\right)=\left\lfloor \frac{B(t)}{B_{\max}}\cdot \left(N_{B}-1\right)\right\rfloor ,$$

where $\hat{B}\left(t\right)\in \left\{0,1,\ldots ,N_{B}-1\right\}$ is the discrete battery state index.

When the battery level falls below the minimum energy required for any transmission, the device is forced to select the silent action P(t)=0, irrespective of the RL agent’s recommendation. This hard constraint prevents the RL agent from recommending infeasible actions and ensures physical validity of the simulation.

### 2.6. Traffic and Queueing Model

Packets arrive at the IoT device according to a Bernoulli process. Let A(t)∈{0,1} be the packet arrival indicator at time slot *t*, where

(11) $$\mathrm{Pr}\left[A\left(t\right)=1\right]=p_{\mathrm{arr}}=0.3.$$

This arrival probability models a moderately loaded IoT sensor that generates data bursts with 30% duty cycle, representative of periodic sensing applications such as environmental monitoring or smart agriculture.

Let Q(t) denote the queue length at the beginning of time slot *t*. The queue evolves according to

(12) $$Q\left(t+1\right)=\min\;\left(Q_{\max},\;\max\;\left(0,\;Q(t)-S(t)\right)+A\left(t\right)\right),$$

where $Q_{\max}$ is the maximum buffer size, S(t)∈{0,1} is the transmission success indicator, and A(t) is the packet arrival indicator.

A packet drop event occurs when the buffer is full and a new packet arrives:

(13) $$\mathrm{Drop}\left(t\right)=1\;\left[A\left(t\right)=1\;\wedge \;Q\left(t\right)=Q_{\max}\;\wedge \;S\left(t\right)=0\right].$$

The average packet sojourn time is related to the time-averaged queue length via Little’s law:

(14) $$\bar{W}=\frac{\bar{Q}}{\lambda_{\mathrm{eff}}},$$

where $\lambda_{\mathrm{eff}}=p_{\mathrm{arr}}\cdot \left(1-\mathrm{Pr}\left[\mathrm{Drop}\right]\right)$ is the effective packet arrival rate. The queue length Q(t) is discretized for the RL state: $\hat{Q}\left(t\right)=\min\left(Q\left(t\right),N_{Q}-1\right)$.

### 2.7. Energy Efficiency and Reward Function

The instantaneous EE at time slot *t* is

(15) $$\mathrm{EE}\left(t\right)=\frac{R(t)}{P(t)+\epsilon },$$

where ϵ≪1 avoids division by zero when P(t)=0.

The composite reward at time slot *t* is

(16) $$r\left(t\right)=\beta_{1}\cdot \mathrm{EE}\left(t\right)-\beta_{2}\cdot Q\left(t\right)-\beta_{3}\cdot \mathrm{Drop}\left(t\right),$$

where $\beta_{1},\beta_{2},$ and $\beta_{3}>0$ are weighting coefficients. The three terms respectively incentivize EE, penalize queue buildup (a proxy for latency), and strongly penalize packet drops. In simulations, we set $\beta_{1}=1$, $\beta_{2}=0.1$, and $\beta_{3}=5$, found via grid search to yield balanced multi-metric performance.

## 3. Reinforcement Learning Formulation

The power control problem is formulated as MDP (S,A,P,R,γ).

### 3.1. State Space


(17) $$s\left(t\right)=\left[\hat{B}\left(t\right),\;\hat{Q}\left(t\right)\right]\in S,\;\left|S\right|=N_{B}\times N_{Q}=100.$$

The state is fully observable by the IoT device. The channel gain h(t) is excluded as it is assumed unavailable before transmission, reflecting practical constraints on simple IoT hardware.

The strict MDP assumption requires that the next state and reward depend only on the current state and action, not on history. Since h(t) is modeled as Independent and Identically Distributed (i.i.d.) across time slots, marginalizing over h(t) at each step yields a well-defined MDP: the expected reward E[r(t)|s(t),a(t)] and the transition distribution $P(s^{\prime }|s,a)$ are both stationary and Markovian, as h(t) is independent of B(t) and Q(t). This is the standard *channel-averaged MDP* treatment for fading channels in model-free RL [17]. The practical cost of excluding h(t) is that the agent cannot condition its power decision on instantaneous channel quality; instead, it learns a policy that is optimal *in expectation* over the channel distribution. As shown in Section 5, the RL agent achieves comparable average throughput to Max-SNR (which has full channel knowledge) while dramatically reducing power consumption, demonstrating that the channel-averaged policy is highly effective. If pilot-based Channel State Information (CSI) was available, adding h(t) to the state would be a straightforward extension.

### 3.2. Action Space


(18) a(t)=P(t)∈P={0,0.1,0.5,1.0}W,|A|=4.

### 3.3. Q-Learning Algorithm

Q-learning learns the optimal action-value function through iterative Bellman updates [24]:

(19) $$Q\left(s,a\right)\leftarrow Q\left(s,a\right)+\alpha \;\left[r\left(t\right)+\gamma \cdot \max_{a^{\prime }}Q\left(s^{\prime },a^{\prime }\right)-Q\left(s,a\right)\right],$$

where α is the learning rate, γ is the discount factor, and $s^{\prime }$ is the next state. An ϵ-greedy policy with exponential decay ensures adequate exploration during training.

### 3.4. Convergence Analysis

Under standard conditions, all state–action pairs are visited infinitely often, learning rate satisfying $\sum_{t}\alpha_{t}=\infty$ and $\sum_{t}\alpha_{t}^{2}<\infty$—Q-learning converges to $Q^{*}\left(s,a\right)$ [24]. In our finite |S|×|A|=400 Q-table, empirical convergence (Q-value changes $<10^{-4}$) is observed within 200–300 training episodes.

### 3.5. Complexity Analysis

Table 1 summarizes the space and time complexity of the proposed Q-learning agent. The Q-table requires only 1.6 KB of SRAM, well within the memory budget of common IoT microcontrollers (e.g., ARM Cortex-M4 with ≥256 KB SRAM). Inference reduces to a single lookup and argmax over |A|=4 entries per time slot: O(1) in the state dimensions and O(|A|) overall. Training is performed offline; the resulting table is deployed as a static lookup, imposing zero online training cost on the device. For a multi-device extension with *N* devices, independent tables scale linearly to $O(N\cdot N_{B}\cdot N_{Q}\cdot |A|)$ space.

## 4. Simulation Setup and Parameter Configuration

Table 2 summarizes simulation parameters. Each experiment is repeated with 10 random seeds; mean values are reported. The coefficient of variation across seeds is below 5% for all schemes. All the simulations are carried out in MATLAB R2025a.

### Benchmark Schemes

Four baseline power control strategies are used for comparison:

Random: $P_{\mathrm{rand}}\left(t\right)∼U\left(P\right)$, serves as a lower bound.

Round Robin: $P_{\mathrm{RR}}\left(t\right)=P\left[t\;\mathrm{mod}\;|P|\right]$, deterministic, channel-agnostic cycling.

Max-SNR (Greedy): $P_{\mathrm{max-SNR}}\left(t\right)=1.0$ W, always transmits at full power; maximizes throughput but ignores battery and efficiency.

Fixed: $P_{\mathrm{fixed}}\left(t\right)=0.5$ W, practical constant-power compromise, representative of legacy IoT deployments without adaptive control.

## 5. Results and Analysis

Before presenting the results, we contextualize the baseline selection. The four benchmark schemes—Random, RR, Max-SNR, and Fixed—represent the four canonical categories of power control: stochastic, deterministic-cyclic, greedy channel-aware, and constant, respectively. These categories are the standard reference points in the RL-based wireless resource management literature [17]. More advanced baselines would require full re-implementation and are addressed qualitatively in Section 5 and identified as the primary future work item.

**Figure 2:**
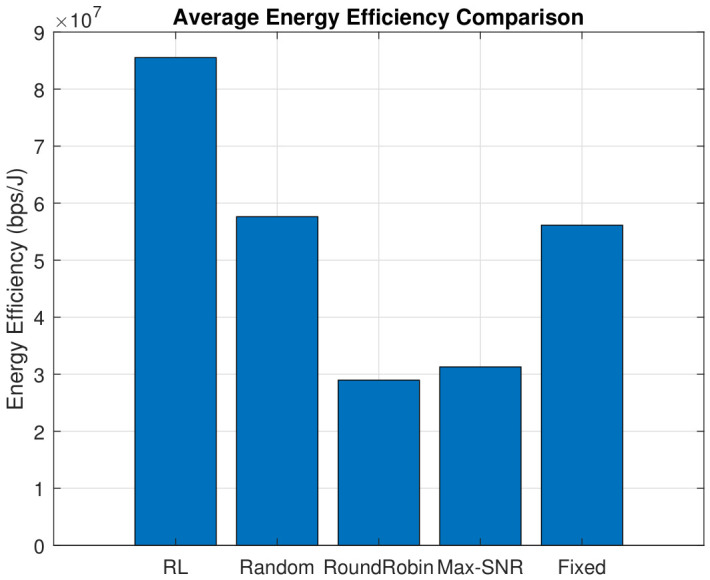
Average energy efficiency comparison of the proposed technique against the state-of-the-art.

Figure 2 presents the overall average EE. The Q-learning-based RL approach achieves the highest average EE at approximately $8.5\times 10^{7}$ bps/J, a 40–50% improvement over the second-best scheme (Fixed at ≈$5.5\times 10^{7}$ bps/J). Random and RR achieve only 2.5–$3.0\times 10^{7}$ bps/J, while Max-SNR reaches $\approx 3.3\times 10^{7}$ bps/J. The superior performance of RL stems from context-dependent power selection: low-to-moderate power in good channels, and silence during deep fades, a strategy no heuristic can replicate.

**Figure 3:**
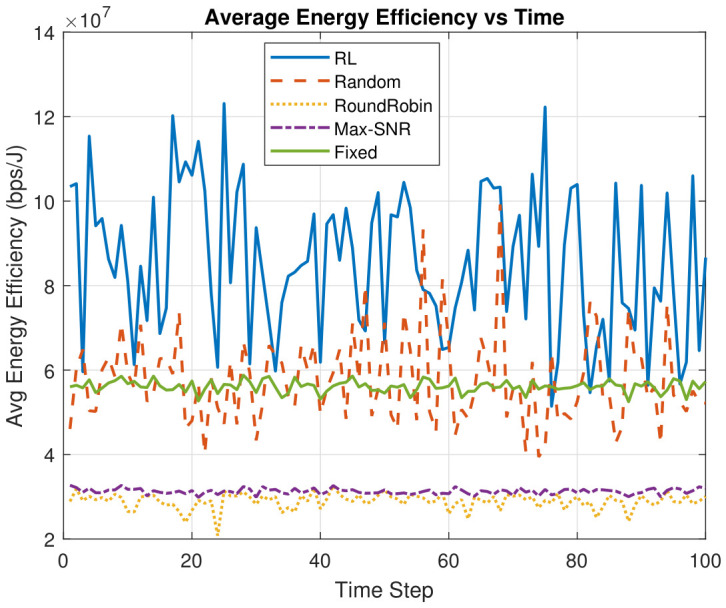
Average energy efficiency versus time.

Figure 3 illustrates the evolution of EE over 100 time steps for the selected strategies. The RL-based policy maintains a relatively stable trajectory, with only moderate fluctuations around its mean performance, indicating that it successfully balances transmission opportunities with energy constraints over time. In contrast, the Random policy exhibits highly erratic behavior, with sharp unpredictable swings in EE due to the lack of any structured decision-making. The Max-SNR strategy, while occasionally achieving high instantaneous EE peaks due to aggressive transmission, suffers from its consistently high power consumption. As the battery depletes more rapidly under this policy, the system is forced into silence more frequently, which significantly lowers its average energy efficiency over time despite the intermittent gains.

**Figure 4:**
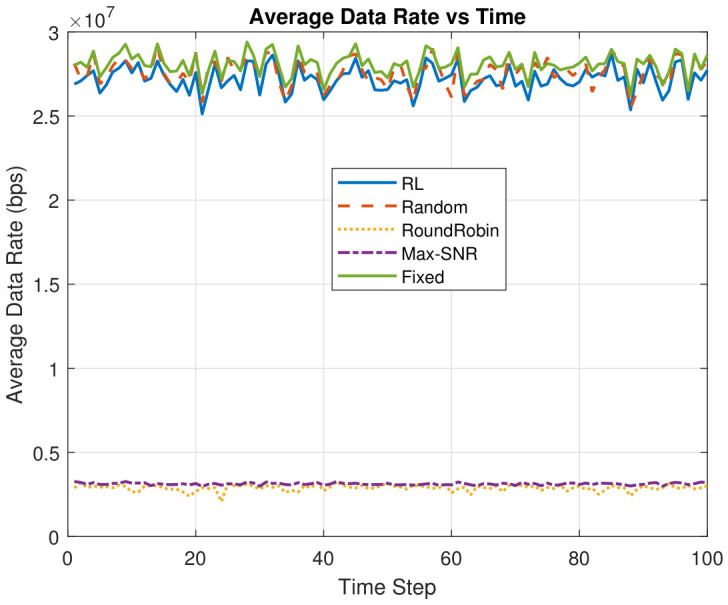
Average data rate versus time.

Figure 4 presents the evolution of system throughput over time for the different strategies. Both the RL-based policy and the Max-SNR scheme achieve comparable average data rates, indicating that the RL agent is able to exploit favorable channel conditions nearly as effectively as the greedy Max-SNR approach. However, a key distinction is that RL attains this performance with significantly lower power expenditure, reflecting a more efficient allocation of transmission resources. The Fixed scheme produces a constant throughput profile, as expected, due to its static transmission policy that does not adapt to channel or battery conditions. In contrast, the RR and Random strategies exhibit noticeable variability and uneven throughput, driven by their lack of channel-awareness and inefficient scheduling decisions. Overall, these results demonstrate that the efficiency gains of the RL approach stem from intelligent power management and adaptive decision-making, rather than any compromise in achievable throughput.

**Figure 5:**
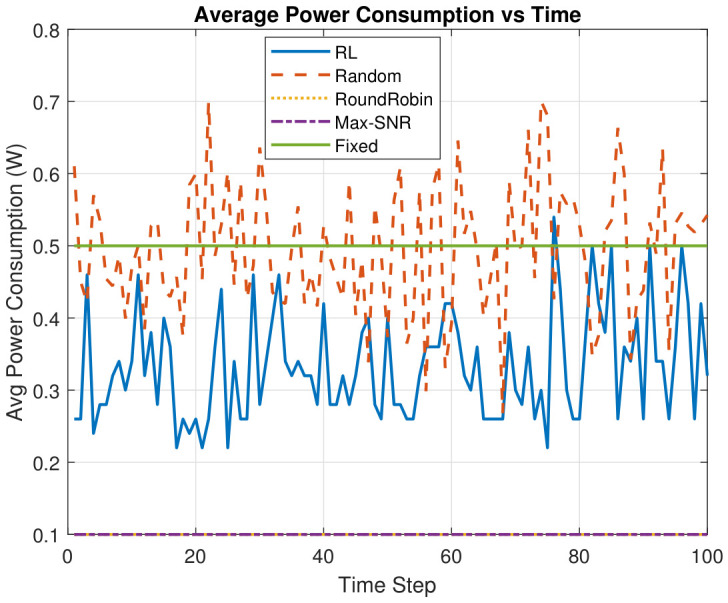
Average power consumption versus time.

Figure 5 illustrates the temporal evolution of power consumption for the considered strategies. The RL-based policy maintains a controlled and adaptive power profile, oscillating between approximately 0.2–0.5 W. This range reflects its ability to dynamically scale transmission power in response to both channel conditions and battery state, resulting in a lower average consumption than all other non-trivial schemes. In contrast, the Max-SNR strategy operates near its maximum power level (close to 1.0 W) for most time steps, prioritizing instantaneous channel quality without regard for energy constraints. This aggressive behavior leads to rapid battery depletion and reduced long-term sustainability. The Random policy, meanwhile, exhibits highly erratic power usage with significant variance, as power levels fluctuate unpredictably due to the absence of any optimization objective. Overall, the moderate and adaptive power profile of the RL approach directly contributes to extending device operational lifetime, while still enabling competitive performance in terms of throughput and efficiency.

**Figure 6:**
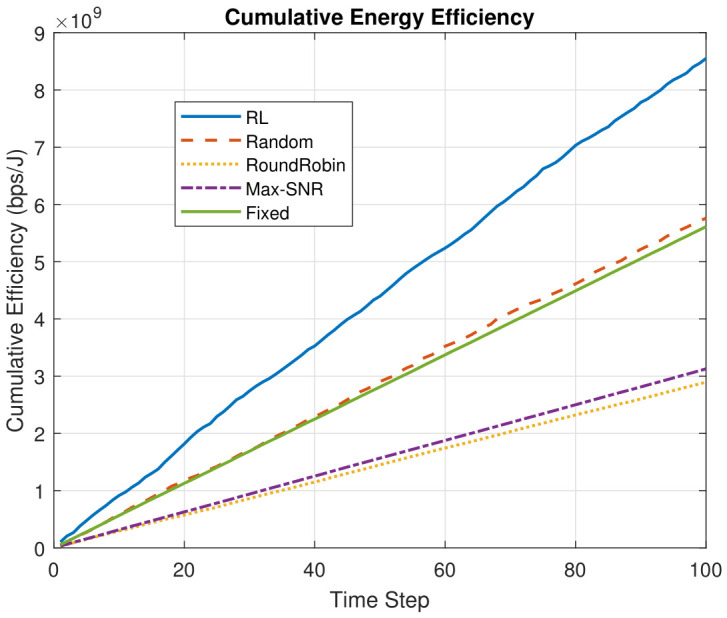
Cumulative energy efficiency over time.

Figure 6 presents the cumulative EE over the 100 time steps, highlighting the long-term performance of each strategy. The RL-based approach exhibits a consistently increasing and smooth trajectory, reaching approximately $8.7\times 10^{9}$ bps/J by the end of the horizon. This corresponds to an improvement of roughly 60% over the Random policy and about 40% over the Fixed scheme. The steady and near-linear growth of the RL curve indicates that it maintains a favorable balance between data transmission and energy consumption throughout the entire duration, without suffering from significant efficiency losses at later stages. In contrast, the curves for Random and Fixed grow at noticeably slower rates, reflecting their less efficient use of energy over time. The steeper slope of the RL curve is particularly indicative of a higher steady-state efficiency accumulation rate, demonstrating that its performance gains are not confined to short-term advantages but persist and compound over time.

**Figure 7:**
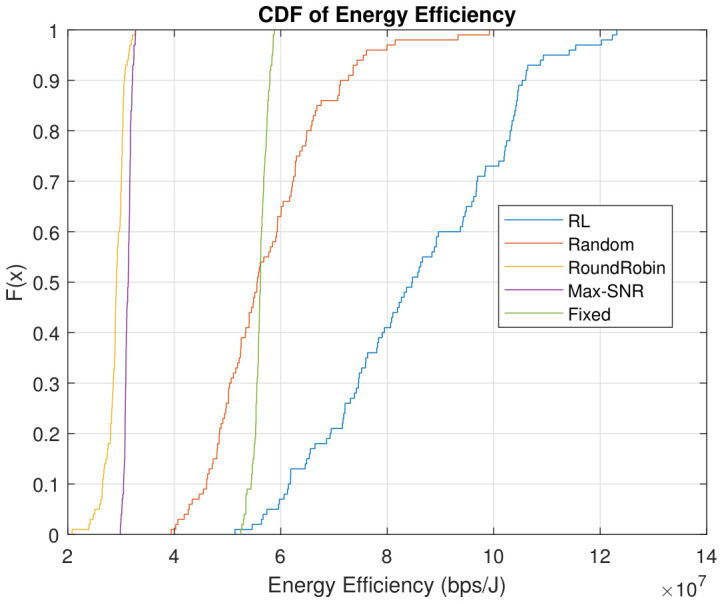
CDF of energy efficiency.

Figure 7 shows the CDF of EE, providing insight into the distribution of efficiency across time steps. The RL-based policy exhibits the most right-shifted CDF, indicating consistently higher efficiency values compared to the other schemes. In particular, more than 50% of the RL time steps achieve an energy efficiency above $7\times 10^{7}$ bps/J, whereas the corresponding median values for the Fixed and Max-SNR schemes are approximately $4\times 10^{7}$ bps/J and $3\times 10^{7}$ bps/J, respectively. This shift highlights the ability of the RL agent to sustain high-efficiency operation for a larger fraction of time. Furthermore, the RL curve appears steeper and more concentrated, reflecting lower variance in achieved efficiency levels. In contrast, the broader spread of the other CDFs indicates greater variability and less predictable performance. The reduced variance of the RL approach is particularly important for practical systems, as it implies more reliable adherence to QoS requirements and fewer instances of performance degradation.

## 6. Discussion, Limitations and Future Directions

Analysis of the trained Q-table reveals three emergent policy characteristics:

1. *Battery-aware conservatism*: At low battery ($\hat{B}\leq 2$), the agent predominantly selects silence or minimum power.
2. *Queue-responsive aggression*: At long queues ($\hat{Q}\geq 7$), the agent escalates to P=0.5 or 1.0 W to clear the backlog and avoid drops.
3. *Moderate power preference*: In most intermediate states, P=0.5 W is favored, matching the peak efficiency region of the Shannon capacity curve at moderate SNR.

DQN achieves richer state representations and handles continuous or high-dimensional state spaces, but requires neural network inference engines unavailable on Class-AB IoT transceivers and is sensitive to hyperparameter tuning. Lyapunov drift-plus-penalty optimization [25] provides provable near-optimal guaranties under stationary channel and traffic statistics, but lacks the adaptivity of model-free RL when statistics are non-stationary; a realistic scenario for HAPS channels subject to atmospheric variation. Tabular Q-learning occupies a deliberate middle ground—hardware-deployable, model-free, and adaptive—at the cost of state-space richness. Quantitative comparison with DQN and Lyapunov-based methods is identified as the primary future work.

### 6.1. Limitations

Key limitations include:

1. The single-device setting isolates the core battery queue EE trade-off without confounding multi-access interference, providing a clean proof-of-concept. Analytically, the robustness to parameter variation can be argued as follows: at lower packet arrival rates ($p_{\mathrm{arr}}<0.3$), the queue rarely builds and the agent’s battery-conserving behavior dominates; at higher rates, the queue-responsive aggression behavior naturally activates. The battery capacity $B_{\max}$ scales the number of transmissions before depletion but does not alter the structure of the learned policy once states are normalized. These arguments support robustness to parameter variations without requiring retraining.
2. The U(0,1) model abstracts Doppler shift, elevation-angle dependency, and atmospheric scintillation. As discussed in Section 1.2, this is a conservative worst-case model; Rician or shadowed-Rician (Loo) models per 3GPP TR 38.811 would be more realistic and constitute a direct future extension.
3. No energy harvesting; battery is non-replenishable in the current model.
4. Stationary channel and traffic statistics; non-stationary environments require periodic retraining.
5. Tabular Q-learning scalability; the curse of dimensionality limits state-space richness.

### 6.2. Future Research Directions

1. Multi-Device Scenarios: Extending to multiple IoT devices sharing the HAPS uplink requires multi-agent RL (MARL) [23].
2. Deep Reinforcement Learning: Replacing the Q-table with a DQN [26] agent enables richer state representations and continuous action spaces. DQN would likely improve performance, particularly in non-stationary channels, at the cost of inference overhead incompatible with current Class-AB IoT hardware [17].
3. Energy Harvesting Integration: Solar or RF energy harvesting transform the problem into a co-optimization of harvest–store–transmit decisions. Lyapunov optimization combined with RL provides a framework with provable near-optimal guarantees [27].
4. Reconfigurable Intelligent Surfaces: Joint optimization of RIS phase shifts and IoT transmit power via RL can substantially improve coverage for shadowed devices [28].
5. Federated RL: With many IoT devices, federated RL enables distributed policy learning without sharing raw data, preserving privacy while leveraging collective device experience [29].
6. Non-Stationary Adaptation: Meta-RL and sliding-window Q-learning can maintain performance under time-varying channel or traffic statistics without full retraining [30].
7. Comparison with Lyapunov-Based Optimization: Lyapunov drift-plus-penalty methods provide provable near-optimal guarantees under stationary channel statistics and represent a strong analytical baseline. Comparison under both stationary and non-stationary conditions would clarify the operating regimes where model-free RL is preferable.

## 7. Conclusions

This paper presented an intelligent and energy-aware RL framework for transmit power control in HAPS-assisted IoT communication systems targeting 6G NTN. The problem was formulated as an MDP with battery level and queue state as the observable state, discrete power levels as actions, and a multi-objective reward function jointly penalizing energy waste, queue buildup, and packet loss. A Q-learning algorithm learns optimal power control policies through direct environmental interaction, without requiring explicit channel modeling. The simulation results within the described single-device i.i.d. channel setting demonstrated 40–50% higher average EE compared to all four benchmarks, alongside lower power consumption, higher packet delivery ratios, and shorter queues. CDF analysis confirmed the RL agent’s statistical reliability under these conditions, with the most right-shifted distribution among all compared schemes. Validation under more realistic channel models (e.g., Rician, 3GPP NTN TR 38.811) and multi-device scenarios remains an important direction for future work.

## Figures and Tables

**Table 1:** Complexity summary.

Metric	Single Device	100-Device Extension
Q-table entries	$400\;(N_{B}\times N_{Q}\times |A|)$	400 per device
Storage	1.6 KB	160 KB total
Inference/slot	O(|A|)=4 ops	O(|A|) per device
Training updates	$O(N_{ep}\cdot T)=50,000$	Offline; loaded as static table

**Table 2:** Simulation parameters.

Parameter	Symbol	Value
Time steps per episode	*T*	100
Training episodes	$N_{\mathrm{ep}}$	500
Max battery level	$B_{\max}$	100 (energy units)
Max queue length	$Q_{\max}$	10 packets
Battery state levels	$N_{B}$	10
Queue state levels	$N_{Q}$	10
Power action space	P	{0,0.1,0.5,1.0} W
Noise power	$N_{0}$	$10^{-9}$ W
Energy scaling	η	10
Arrival probability	$p_{\mathrm{arr}}$	0.3
Learning rate	α	0.1
Discount factor	γ	0.95
$\left(\epsilon_{\max},\epsilon_{\min}\right)$	-	(1.0,0.01)
Reward weights	$\left(\beta_{1},\beta_{2},\beta_{3}\right)$	(1,0.1,5)
Regularization	ϵ	$10^{-6}$

## Data Availability

The simulation results and configuration details supporting the findings of this study are available from the corresponding author upon reasonable request.
